# Alignment of the Measurement Scale Mark during Immersion Hydrometer Calibration Using an Image Processing System

**DOI:** 10.3390/s131114367

**Published:** 2013-10-24

**Authors:** Luis Manuel Peña-Perez, Jesus Carlos Pedraza-Ortega, Juan Manuel Ramos-Arreguin, Saul Tovar Arriaga, Marco Antonio Aceves Fernandez, Luis Omar Becerra, Efren Gorrostieta Hurtado, Jose Emilio Vargas-Soto

**Affiliations:** 1 National Metrology Center (CENAM), km 4.5 Carretera a los Cues, Mpio. El Marques, Queretaro 76241, Mexico; E-Mails: lpena@cenam.mx (L.M.P.-P.); lbecerra@cenam.mx (L.O.B.); 2 Informatics Faculty, Autonomous University of Querétaro, Avenida de las Ciencias s/n, Juriquilla, Queretaro, Qro. C.P. 76230, Mexico; E-Mails: jramos@mecamex.net (J.M.R.-A.); saulotov@yahoo.com.mx (S.T.A.); marco.aceves@gmail.com (M.A.A.F.); efrengorrostieta@gmail.com (E.G.H.); emilio@mecatronica.net (J.E.V.-S.)

**Keywords:** hydrometer calibration, image processing, Cuckow's method

## Abstract

The present work presents an improved method to align the measurement scale mark in an immersion hydrometer calibration system of CENAM, the National Metrology Institute (NMI) of Mexico, The proposed method uses a vision system to align the scale mark of the hydrometer to the surface of the liquid where it is immersed by implementing image processing algorithms. This approach reduces the variability in the apparent mass determination during the hydrostatic weighing in the calibration process, therefore decreasing the relative uncertainty of calibration.

## 1. Introduction

Hydrometers are widely used instruments in industry to measure the density of liquids. Depending on the application, the hydrometer may change its name, *i.e.*, alcoholmeter to measure the percent of alcohol in breweries [1–3]; Brix hydrometer [4], to measure the percent of sugar in sugar cane solutions; lactometers, to determine the fat content from milk density [5] and API hydrometers or thermo-hygrometers [6], but in the end, no matter in which units the hydrometer is graduated, it measures the density of a liquid [7]. These instruments are generally manufactured in glass, having two main parts: the body, sometimes with a bulb and a hydrometer stem. The body has a cylindrical shape with a load in the bottom; the hydrometer stem is a thin hollow tube attached to the upper portion of the body. A paper with a graduated scale is fixed inside the hydrometer stem. The hydrometer floats vertically on the liquid where it is immersed by means of the Archimedes' principle; the density of the liquid is the reading of the scale mark at the surface level of the liquid.

Cuckow's method is used for the calibration of hydrometers [8]. It consists in obtaining the mass of the hydrometer both in air and when it is submerged to a desired point of its scale mark, in a liquid of known density (hydrostatic weighing).

One of the difficulties in calibration by Cuckow's method, when the hydrometer is immersed in the liquid of known density, is how to align the desired scale mark of the hydrometer with the liquid surface because of the meniscus formed around the hydrometer stem caused by the surface tension of the liquid. This alignment depends on the skill of the metrologist, giving rise to human errors that have a direct impact in the best estimate and in the uncertainty of the calibration result. Previous work has been done in order to minimize this source of error by means of semi-automated [9] or automated [10,11] systems based on image processing acquisition.

A methodology based on a vision system and image processing algorithm developed at CENAM is proposed to improve both the alignment of the desired scale mark at the same level of the surface liquid, and the resolution of the hydrometer during its calibration. Experimental results show the improvement to the calibration of hydrometers with the vision system alignment method *vs.* the traditional alignment method, *i.e.*, with the naked eye or with a magnifying glass. Also, key and supplementary international comparisons have been carried out to validate the methodology proposed in this work.

## 2. Hydrometer Calibration System

**Figure 1.**
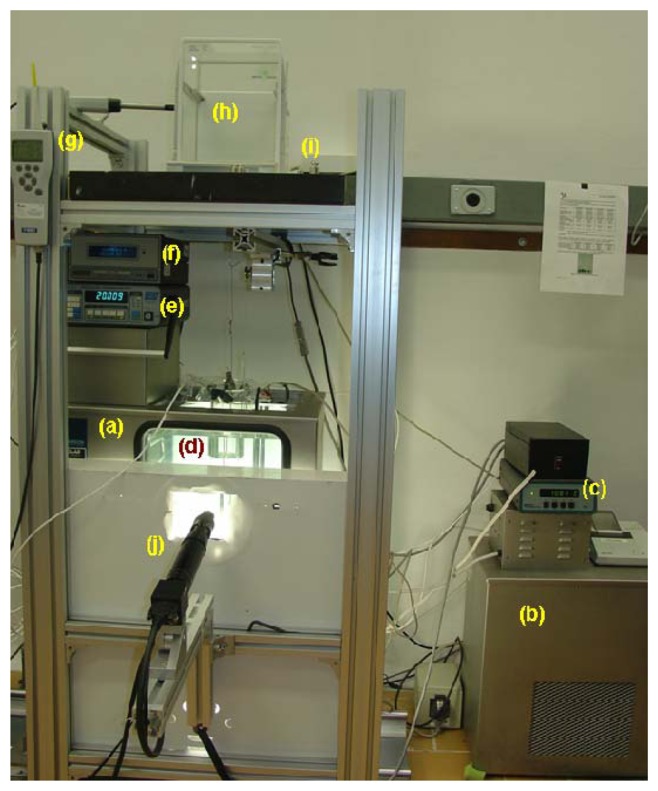
Hydrometer Calibration System: (**a**) thermostatic bath; (**b**) external cooler; (**c**) external temperature controller; (**d**) glass vessel containing the reference liquid; (**e**) thermometer for measuring the liquid temperature; (**f**) barometer; (**g**) thermo-hygrometer; (**h**) weighing instrument (balance); (**i**) mass standards.

The system that has been developed in CENAM for hydrometers calibration, includes a thermostatic bath with an external temperature controller, a glass vessel to contain the reference liquid, a platinum resistance thermometer to measure the temperature of the liquid, an hygro-thermometer, a barometer to obtain the density of air, and a set of mass standards and a weighing instrument (balance) capable to perform hydrostatic weightings, as can be seen on Figure 1.

Usually, hydrometers are calibrated at three or four graduation marks of the scale and for each of them the correction *C_ρn_* can be calculated as *C_ρn_* = *ρ_x_* − *ρ_n_*, where *ρ_x_* is the density of the liquid in which the hydrometer would freely float at the scale mark *ρ_n_* [12]. Hence, the measurand in hydrometers calibration is the correction *C_ρ_n__* [13].

The mathematical model employed in hydrometer calibration (Cuckow's Method) to obtain *ρ_x_* is:

(1) $$\rho_{x}=\left(\rho_{L}\left[1+\alpha \left(t_{L}-t_{0}\right)\right]-\rho_{a1}\left[1+\alpha \left(t_{a}-t_{0}\right)\right]\right)\cdot \left[\frac{m_{a}+\frac{\pi D\gamma_{x}}{g}}{m_{a}-m_{L}+\frac{\pi D\gamma_{L}}{g}}\right]+\rho_{a1}\left[1+\alpha \left(t_{a}-t_{0}\right)\right]-\varepsilon_{d}$$

with:

(2) $$m_{a}=m_{p1}\left(1-\frac{\rho_{a1}}{\rho_{p1}}\right)+\Delta m_{1}-\varepsilon_{d(\text{bal})}$$

(3) $$m_{L}=m_{p2}\left(1-\frac{\rho_{a2}}{\rho_{p2}}\right)+\Delta m_{2}+C_{g}-\varepsilon_{d(\text{bal})}$$

(4) $$C_{g}=\frac{m_{p2}}{g}\frac{\partial g}{\partial h}\Delta h$$

where:
*ρ_x_*density of the hydrometer at selected scale mark x.*ρ_L_*density of reference liquid.*ρ_a1_*density of air during mass determination of hydrometer in air (*m_a_*).*m_a_*mass of the hydrometer in air.*m_L_*apparent mass of hydrometer immersed in the liquid up to scale mark *x*.*g*acceleration due to local gravity.*π*Pi value = 3.14159265.*D*diameter of the hydrometer stem at the selected scale mark *x*.*γ_x_*surface tension coefficient of the liquid where hydrometer is used.*γ_L_*surface tension coefficient of reference liquid.*α*thermal expansion coefficient of hydrometer (usually glass).*t_L_*temperature of reference liquid.*t_0_*nominal temperature value of hydrometer's scale.*ε_d_*error due to hydrometer's resolution.*m_p1_*mass standard used during mass determination of hydrometer in air.*m_p2_*mass standard used during apparent mass determination of hydrometer immersed in liquid.*ρ_p1_*density of mass standard used during mass determination of hydrometer in air.*ρ_p2_*density of mass standard used during apparent mass determination of hydrometer immersed in liquid.*ρ_a2_*density of air during apparent mass determination of hydrometer in liquid (mL).*Δ_m1_*mass difference during weighings in air.*Δ_m2_*mass difference during weighings in liquid.*C_g_*gravity correction due to difference in centre of mass.*ε_d(bal)_*error due to resolution of the balance.*∂g*/*∂h*vertical gravity gradient.

To obtain the mass both in air and in liquid of the hydrometer, the simple substitution method is used; the mass m_a_ for weightings in air, and apparent mass m_L_, when the hydrometer is immersed in the liquid, are compared against mass standards using the balance as a comparator. The alignment of the desired point of scale mark at the horizontal level of the reference liquid is a difficult task, because the meniscus formed around the hydrometer stem due to the surface tension of the liquid hides the scale mark. The operator performs manually this alignment using a magnifying lens and reading the scale mark below the surface of the liquid, introducing human errors depending on the operator's skill, sight and experience. The contribution to the uncertainty due to repeatability in the apparent mass determination in the liquid *Δm_2_* is highly significant.

## 2.1. The Vision System

**Figure 2.**
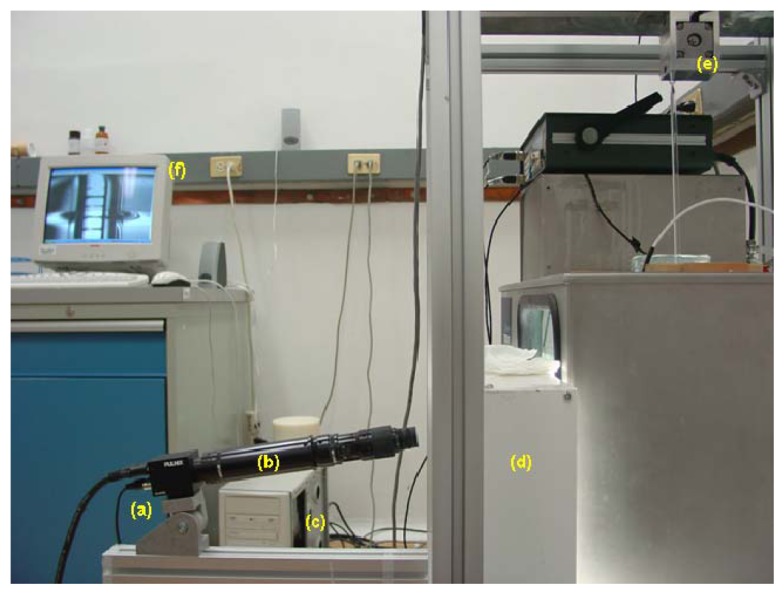
Vision system adapted to the hydrometers calibration: (**a**) camera; (**b**) lens array; (**c**) frame grabber installed in the PC; (**d**) light source; (**e**) motorized-sinker; (**f**) image displayed in the monitor of the scale marks of the hydrometer immersed in liquid.

In order to reduce this human error, the vision system which is shown in Figure 2 was adapted to the actual hydrostatic weighing system consisting of a high resolution (1,300 H × 1,030 V pixels) PULNIX TM-1320-15CL monochromatic CCD camera (Pulnix Imaging Products, Sunnyvale, CA, USA), a lens array, an 8-bits NI-PCI-1428 frame grabber camera link (National Instruments, Austin, TX, USA) and a light source. Also, a glass sinker controlled by a stepper motor was installed to adjust the level of the reference liquid. The level of the liquid will rise (or fall) when the sinker is immersed (or emerged) in the liquid. The motorized-sinker is used for the finest adjustment of the scale mark to the surface of the liquid.

**Figure 3.**
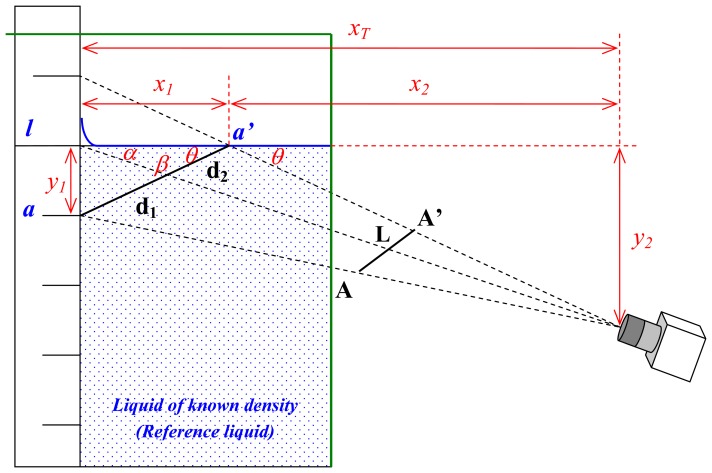
Scheme based on the pin-hole camera model approach to obtain the relation between *d_1_* and *d_2_* (*K* value).

By using the vision system, it is possible to acquire images of the scale mark of the hydrometer at the level of the liquid; the frame grabber converts the images from an analog signal to digital format for future image processing. The camera is located at an angle *θ* below the horizontal level of the liquid, as can be seen on the scheme presented on Figure 3, so the target mark *L* can be seen. The mark of the scale below the target mark *A* is reflected on the surface of the liquid, so there is a virtual image of marks *A* and *A*'. The alignment of *L* at the surface level is accomplished when the distance in pixels between *A* and *L* and that between *L* and *A*' is the same at the image plane. However, due to the position of the camera, corrections of these two distances should be made [9]. The marks *L, A* and *A*' on the hydrometer scale will be presented later, in the alignment using image processing section.

In order to obtain the relation *K* between the distances from *A* to *L* (*d_1_*) and form *L* to *A*' (*d_2_*) a pin-hole camera model approach has been employed as shown in Figure 3. The relation between *d_1_* and *d_2_* is as follows:

(5) $$\frac{d_{2}}{d_{1}}=K$$

*K* is obtained by means of the following equation:

(6) $$K=\frac{1}{\frac{(\sin\beta )\cdot \sqrt{{x_{1}}^{2}+{y_{1}}^{2}}}{x_{1}\;\cdot \;\sin\alpha }-1}$$

with:

(7) β=180°−α−θ

(8) $$\alpha =\arctan\left(\frac{y_{2}}{x_{T}}\right)$$

(9) $$\theta =\arctan\left(\frac{y_{1}}{x_{1}}\right)$$

(10) $$x_{1}=\frac{x_{T}}{\frac{y_{2}}{y_{1}}+1}$$

The known values that can be measured are the horizontal distance from the hydrometer stem to the camera (*x_T_*), the vertical distance (*y_1_*) between two consecutive marks of the hydrometer (usually the mark to be aligned and the mark below immersed in the liquid), and the vertical distance from the liquid surface to the camera (*y_2_*).

### 2.2. Image Processing Algorithm

**Figure 4.**
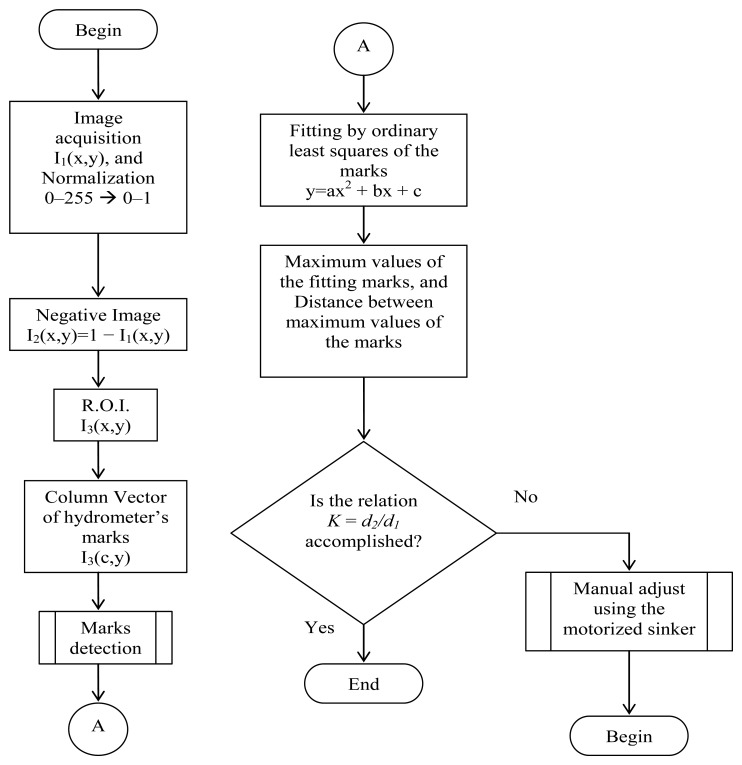
Image processing algorithm flowchart.

The step by step procedure of the image processing algorithm developed to perform the alignment of the hydrometer's calibration scale mark at the surface level of the reference liquid is shown in Figure 4.

Once the image *I_1_(x,y)* is obtained, a pre-processing procedure is performed by normalizing the gray levels from integer values of 0–255 to double floating point values in the range of 0–1, and inverting the normalized image (negative image) by applying the following equation:

(11) $$I_{2}\;\left(x,y\right)=1-I_{1}\;\left(x,y\right)$$

From *I_2_(x,y)* the region of interest (ROI), which includes the calibration mark, the mark below the reference mark and its reflection on the surface of the liquid, is selected. Afterwards, a procedure to detect the three marks is applied to a column vector of the ROI, which includes a noise reduction subroutine, where information of pixel position against gray level is relevant. A plot of the data obtained after this procedure shows that each scale mark can be approximate to a second order equation by ordinary least square fitting. The pixel position at maximum gray level for each mark is obtained: *p_1_* for the pixel position of the mark below the calibration mark, *p_2_* for the pixel position of the calibration mark, and *p_3_* for the virtual image (the reflection) of the mark below the reference mark. Then, the distance *d_1_* and *d_2_* are calculated using the values *p_1_*, *p_2_* and *p_3_* as follows:

(12) $$d_{1}=\left|p_{1}-p_{2}\right|$$

(13) $$d_{2}=\left|p_{3}-p_{2}\right|$$

with *d_1_* and *d_2_* the *K* relation is calculated. If the calculated *K* value is not equal to the expected *K* value, the motorized sinker is used to adjust the liquid level so the value of *d_2_* changes until the *K* value is obtained, due to the fact that *d_1_* remains equal.

## 3. Experimental Section

The majority of primary metrology laboratories around the world use the Cuckow method to calibrate the immersion hydrometer, due to the fact that its main advantage is the use of only a standard liquid of known density to calibrate the equipment for any measurement interval.

The procedure described in previous section proposed that an alignment mark in the hydrometer scale can be calibrated utilizing digital image processing, which considers that the criterion values *K_calc_* and *K_meas_* are equal within a confidence interval.

**Figure 5.**
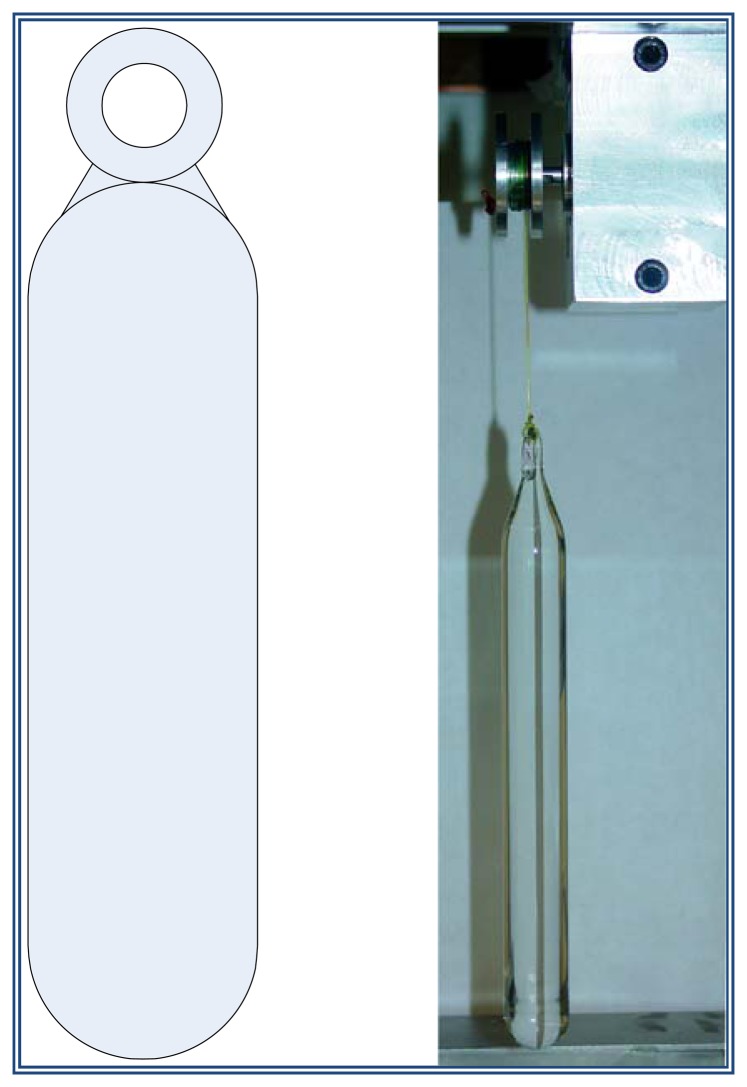
Sinker tied to a stepper motor.

In case that the alignment doesn't occur, it is necessary to immerse or emerge the hydrometer shaft as required in the reference liquid. This occurs by the use of a “sinker” made of a material which does not react chemically with the reference liquid, because if this occurs its density value could change. To avoid the, the material chosen to fabricate the “sinker” was solid borosilicate. The sinker's dimensions are 172.75 mm long and 177.0 mm in diameter. In addition, the sinker has an eyelet so it can be hung. Figure 5 shows a photograph of the sinker.

### 3.1. Adjustment in the Liquid Level Using a Motorized Sinker

**Figure 6.**
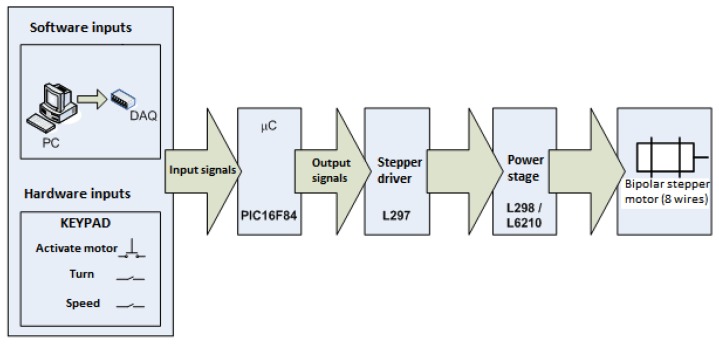
Open loop controller of the stepper motor.

The sinker of Figure 5 is utilized to vary the liquid level by immersion. This solid is held by the grommet with a nylon thread which is wound on the pulley of a stepper motor whose movement is manually controlled using a keypad. To control the stepper motor movement, an open loop controller was implemented in the electronic circuit as shown in the block diagram of Figure 6.

The stepper motor is a brushless synchronous electric motor with the ability to divide a full rotation into a large number of steps. Each step represents a small discrete angular movement. The advantage of this motor is that it can be used for position control without feedback. For controlling movement of the stepper motor a PIC16F84A microcontroller (Microchip Technology, Inc., Chandler, AZ, USA) was selected. This microcontroller satisfies the needs for this application. The microcontroller has two ports that can be configured as either inputs or outputs. For this application five pins of port A were configured as an input and the eight pins of port B were configured as an output.

Both ports handle incoming and outgoing digital Transistor-Transistor Logic (TTL) signals. To send digital signals from the microcontroller, we designed an electronic circuit, which can receive digital signals produced by a keypad (hardware) or by sending digital signals from an acquisition card data (DAQ) installed in a personal computer (PC).

Signals from the keypad are sent to the port A of the microcontroller, which are processed by the algorithm programmed into its memory and making decisions to enable or disable the output signals through the pins of port B.

The output signals of the microcontroller are sent to an L297 integrated circuit (STMicroelectronics, Geneva, Switzerland) which works as a driver or interpreter between microcontroller and the power to move the stepper motor. The L297 has the ability to “translate” the digital input signals in the correct sequence of pulses required to be sent to the power amplifier (L298 and L6210 integrated circuits, STMicroelectronics) to properly energize the motor windings and get their movement.

The L297 circuit has as an additional function of cutting the dual PWM circuit which serves to regulate the current flowing through the motor windings.

**Figure 7.**
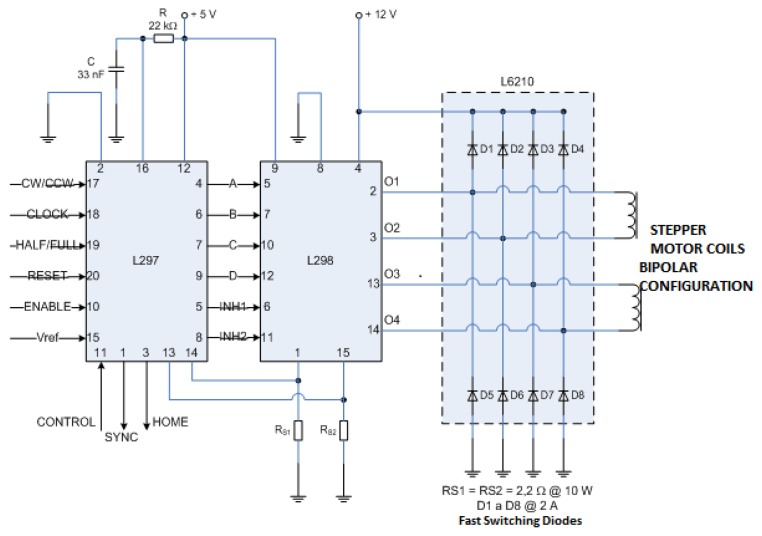
Connection diagram between the L297, L298 and the stepper motor windings.

The circuit L297 is connected to a L298 integrated circuit, which internally has a dual H-bridge transistors designed for higher voltage and current values of electrical current through their TTL digital inputs, it can handle inductive loads such as solenoids, relays, DC motors and stepper motors. The emitters of the transistors of the lower H-bridge are connected together and the respective output terminal is used for connecting an external resistor to sense the current flowing through the coil (RS1 and RS2). It uses a separate power supply for making that the circuit logic works at a lower DC voltage. It also has two pins to enable operation of each of the H-bridges. So the motor windings are discharged rapidly during the transition from one sequence to the next to energize, it uses a Schottky fast recovery diode array type capable of maintain peak currents up to 2 A, the diode array is encapsulated in the integrated circuit L6210. Figure 7 shows the connection diagram of integrated circuits L297, L298 and L6210 to the motor windings. The stepper motor used to move the sinker is an Applied Motion brand HT17-075 (Applied Motion Products, Watsonville, CA, USA) model with a resolution of 1.8° per step.

To carry out the rise and fall of the shaft of the hydrometer immersed in reference liquid, the borosilicate sinker is also immersed in the liquid. The upper end of the sinker is attached through its eyelet with hemp yarn which is wound on a pulley fixed to the shaft of the stepper motor. The motor, when rotated clockwise (CW), causes the sinker go down and soak in the liquid causing the liquid level rise due to the volume of liquid displaced by the sinker gradual immersion, the resulting effect is also the shaft becomes immersed into the liquid. Conversely, when the motor rotates counterclockwise (CCW), the sinker rises and reference fluid emerges causing the fluid level down and making the hydrometer shaft also remove reference fluid.

### 3.2. Experimentation

For the hydrometer calibration, once the mass in the air is measured and the points of the scale to be calibrated are selected, the hydrometer is immersed in the reference liquid. Previously, the fluid temperature is set and control referenced to the same temperature specified in the hydrometer scale. For the immersion in the liquid, the hydrometer is held in the highest part of the shaft, which is fixed to a device that serves as a mechanical suspension whose length can be adjusted manually, then, it can be hanged beneath the weighing pan of the balance. During of immersion of the hydrometer in the reference liquid, it should be avoided getting wet the stem above the first calibration point, which is the lowest point in the scale. It must be left two hours for thermal stabilization before starting the measurements.

After the thermal stabilization period, adjustment in the suspension height must be carried out to manually locate by sight the point of the hydrometer scale at the same level of the liquid. From here, a vision system operates, which is adjusted by zooming and focus to see on the images three interest marks labeled as *A*, *L* and *A*'.

Later, a program acquires an image from the vision system, select the region of interest (ROI) covering the three marks of the hydrometer. Then a column vector is selected at the center of the ROI to detect the marks, and a least squares fitting is performed to fit a parabola of the marks; by doing this, the maximum points of the parabolas select the maximum can be used to measure the distances among them, to get the *K_meas_* value and compare it with the *K_calc_* value. In case that these values are not equal, the keypad is used to move the sinker immerse in the liquid on the adequate direction to adjust the liquid level, achieving that the *d_2_* distance change in respect to the position of mark *A*' (reflection of the mark A of the liquid surface).

**Figure 8.**
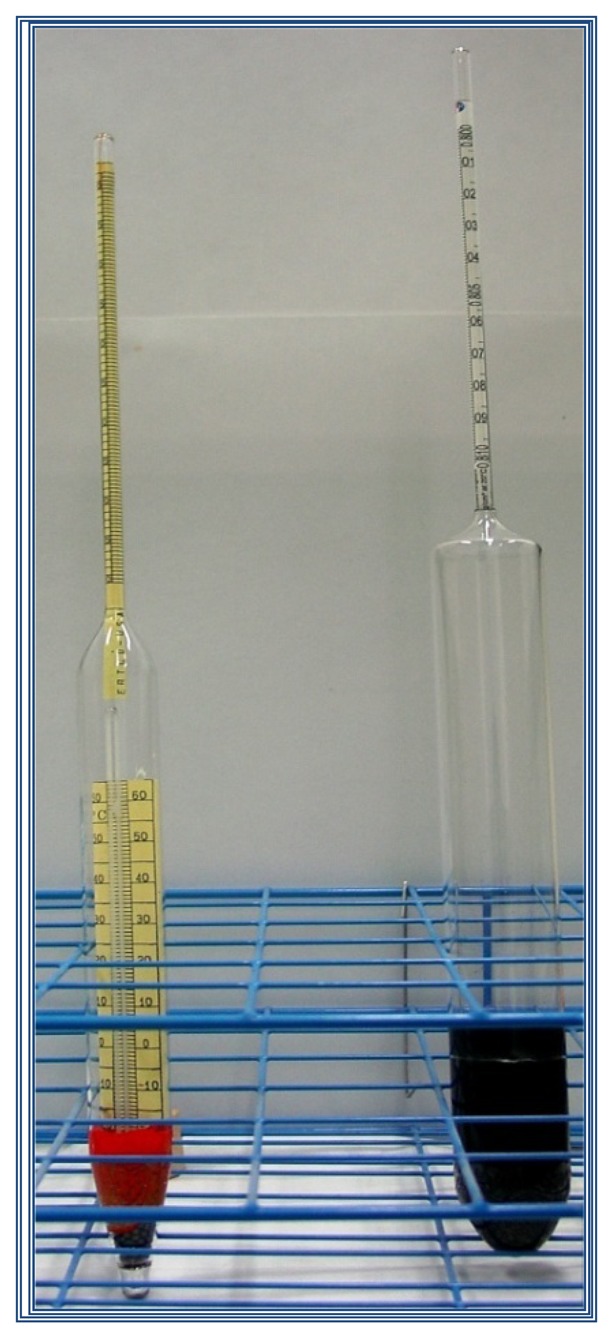
Hydrometers used in the experimentation.

To demonstrate the difference in the alignment process using the traditional method against the method with the vision system and the impact it has on the calibration uncertainty, a calibration was conducted with two hydrometers with different metrological characteristics, the first one is a high accuracy hydrometer and the second one is a medium accuracy hydrometer. Both hydrometers are commonly used in the industry for determining the density of liquids and are presented in Figure 8.

Both hydrometers were calibrated at the midpoint of the scale, using two methodologies: the traditional calibration method (visual alignment), and calibration by the vision system method (semi-automatic alignment) [10,12].

**Figure 9.**
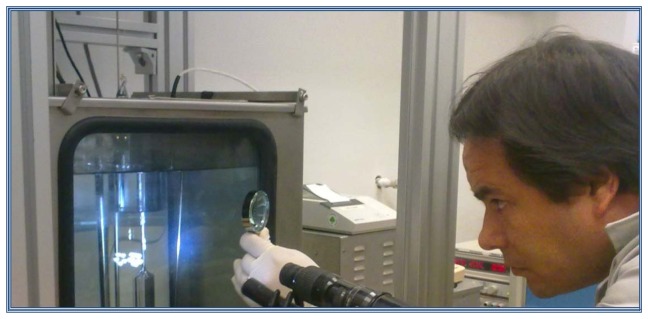
Traditional hydrometer calibration process.

*Traditional calibration.* The first calibration was done with the old method, *i.e.*, using a magnifying glass and left to the judgment of metrologist to mark the location of the scale to calibrate the reference surface of the liquid. This procedure is carried out as can be observed on Figure 9.

**Figure 10.**
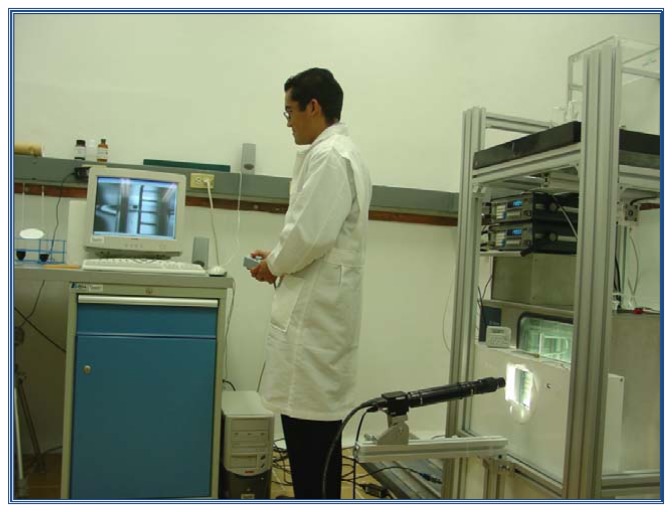
Calibration using the vision system.

*Calibration with the Vision System.* The second calibration was performed using the vision system and image processing algorithm explained in previous sections and it can be observed on Figure 10.

During the traditional calibration method, images of the hydrometer immersed in reference liquid were taken with the vision system, and once the metrologist considered it under assessment, the calibration mark was aligned to the level of the liquid surface. These images are analyzed using the same algorithm to determine the distances between the marks of interest and get *K_meas_* factor value. Tables 1 and 2 show the characteristics of the hydrometers that were calibrated using both methods:

For both high and medium accuracy hydrometers the calibration using the vision system the *K_calc_* was determined with the approximation of the system presented in Figure 3, measuring the distances *x_T_*, *y_1_* and *y_2_*, and using Equations (6, 7, 8, 9, 10). The results are presented in Tables 3 and 4.

The *K_calc_* values have an associated uncertainty of 0.03 with a coverage factor of *k* = 2. This value is estimated applying the Guide to the expression of Uncertainty in Measurement (GUM) [14] to the mathematical models of Equations (6–10) in the pinhole model approach.

### 3.3. Results

In Section 2 was explained that the measurand in hydrometers calibration is the correction in the mark of its scale, *C_ρ_n__*. Cuckow's method requires two measurements of the hydrometer; the first is the mass determination in air *m_a_*, and the second is the mass determination in the liquid *m_L_* when the hydrometer is immersed in the reference liquid until the calibration point of its scale.

The results of the measurements made to both hydrometers consider the next parameters: determination of the air mass *m_a_*, determination of the liquid mass *m_L_*, correction of the calibration scale point *C_ρ_n__*, its uncertainty *u*(*C_ρ_n__* ), and finally the alignment using the proposed image processing algorithm.

The vision system and image processing algorithm developed in this work are used to align the mark on the hydrometer scale at the reference liquid surface during the determination of the mass in the liquid *m_L_*.

For calibration of each hydrometer, six AB weighing cycles scheme were performed using the calibrated set of weights and the weighing instrument (balance) as mass comparator to determine the mass in air *m_a_* and the mass when the hydrometer is immersed in the liquid reference *m_L_*. A represents the mass value of the calibrated weights, and B represents the sample, that is, the hydrometer. The measurements for the determination of the mass in the air for both hydrometers are presented in Table 5.

As in the determination of the mass in air, the mass when the hydrometer is immersed in the reference liquid *m_L_* is obtained by comparison against standard weights performing six AB weighing cycles. Table 6 presents the measurements performed with the balance to get the *m_L_* value and its standard deviation σ*m_L_* for the high accuracy hydrometer (*d* = 0.1 kg/m^3^). On the left side of Table 5, the values obtained with the traditional alignment method are shown, and on the right side, the values obtained with the vision system are shown.

For the hydrometer of medium accuracy (*d* = 0.5 kg/m^3^) the measurements and results of *m_L_* and σ*m_L_* using both traditional and vision system alignment methods are shown in Table 7.

From the measurement it can be seen that for the case of the high-accuracy hydrometer, the standard deviation obtained with the traditional alignment method is approximately 15 times the standard deviation obtained with the alignment method of the vision system and image processing algorithm; in the case of the medium accuracy hydrometer is about 71 times greater.

On Table 8 the results of calibration of the high accuracy hydrometer are presented (*d* = 0.1 kg/m^3^) at the point *ρ_n_* = 805 kg/m^3^, the values were obtained with both the traditional and the vision system alignment methods.

Table 9 show the results of the calibration with both traditional and vision system alignment methods for the calibration point *ρ_n_* = 1,025 kg/m^3^ from the medium accuracy hydrometer (*d* = 0.5 kg/m^3^).

#### 3.3.1. Uncertainty Budget

As was explained in Section 2, the measurand in the calibration of hydrometers is the correction *C_ρ_n__* that must be applied to the selected calibration point *ρ_n_* of the hydrometer scale. This correction is obtained using Equation (14):

(14) $$C_{\rho_{n}}=\rho_{x}-\rho_{n}$$

where *ρ_x_* is the liquid density where the hydrometer freely float immersed at the mark *ρ_n_*, also, the *ρ_x_* value is obtained by calibration using the Cuckow's method given by Equation (15):

(15) $$\rho_{x}=\left(\rho_{L}\left[1+\alpha \left(t_{L}-t_{0}\right)\right]-\rho_{a1}\left[1+\alpha \left(t_{a}-t_{0}\right)\right]\right)\cdot \left[\frac{m_{a}+\frac{\pi D\gamma_{x}}{g}}{m_{a}-m_{L}+\frac{\pi D\gamma_{L}}{g}}\right]+\rho_{a1}\left[1+\alpha \left(t_{a}-t_{0}\right)\right]-\varepsilon_{d}$$

Applying the GUM method [12] (see Equation (18)), the uncertainty of the correction of the hydrometer immersed at the scale mark *ρ_n_* is:

(16) $$u\left(C_{\rho_{n}}\right)=\sqrt{u^{2}\;\left(\rho_{x}\right)+u^{2}\;\left(\rho_{n}\right)}$$

Due to the fact that *ρ_n_* is a constant value (that is a nominal value from the scale point of the hydrometer under calibration, a constant value has no uncertainty) then the correction uncertainty is reduced to:

(17) $$u\left(C_{\rho_{n}}\right)=u\left(\rho_{x}\right)$$

**Figure 11.**
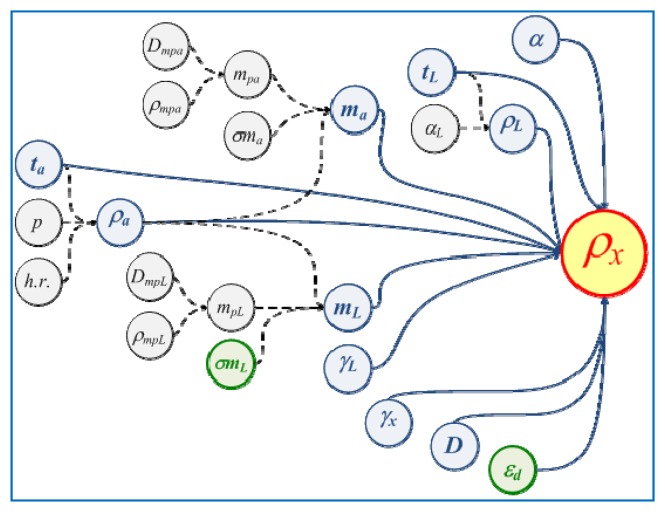
Tree diagram of the uncertainty contributions in the calibration of immersion hydrometers by Cuckow's method.

According to the GUM and applying the uncertainty propagation law to the mathematical model of Equation (15), the correction uncertainty is:

(18) $$u\left(C_{\rho_{n}}\right)=u\left(\rho_{x}\right)=\sqrt{\overset{n}{\underset{i=1}{\text{∑}}}{\left[\frac{\partial \rho_{x}}{\partial \rho_{xi}}u\left(x_{i}\right)\right]}^{2}}$$

where:

$\frac{\partial \rho_{x}}{\partial x_{i}}$ is the sensitivity coefficient of the input quantity *x_i_*
*u* (*x_i_*) is the uncertainty of the input quantity *x_i_*
The input quantities *x_i_* for *ρ_x_* are shown in Figure 11.

It can be observed from Figure 11 that the input values depend on other secondary variables like the reference liquid density *ρ_L_*, depends on the thermal expansion coefficient of the liquid *α_L_* and of the temperature of the liquid *t_L_*. Also in Figure 11, the main variables are distinguished with circles and blue font and the secondary variables in circles and black font. Secondary variables are described as:
*α_L_*:Thermal expansion coefficient of the reference fluid (pentadecane).*m_pa_*:Mass of the standards used to determine the air mass in the hydrometer, *m_a_*.*σm_a_*:Standard deviation of the measurements to get the mass in the air.*ρm_pa_*:Density of mass standards used to determine *m_a_**Dm_pa_*:Drift of mass standards used to determine *m_a_**m_pL_*:Mass of the standards used to obtain the mass in the hydrometer's liquid, *m_L_**ρm_pL_*:Mass standards density used in determine *m_L_**Dm_pL_*:Drift of mass standards used to determine *m_L_**p*:Air barometic pressure during the calibration*h.r.*:Air relative humidity during the calibration

The uncertainty contributions *ε_d_* and *σm_L_* are distinguished in green font in Figure 11, those contributions corresponds to the scale division error or hydrometer resolution and also to the standard deviation in the measurements to obtain the mass of the hydrometer immersed in the liquid until the calibration point of the stem *ρ_n_*. These contributions are reduced significantly by using the vision system and the calibration mark alignment algorithm introduced in this work. Table 10 shows the uncertainty budget calibration with the traditional alignment method, and Table 11 shows the uncertainty budget calibration using the vision system method together with the image processing algorithm. Both calibrations were applied to the high accuracy hydrometer (*d* = 0.1 kg/m^3^).

The same procedure was applied for the medium accuracy hydrometer (*d* = 0.5 kg/m^3^). Results for the uncertainty budget calibration with the traditional alignment method are shown in Table 12. The uncertainty budget calibration using the vision system method together with the image processing algorithm is shown in Table 13.

Considering the uncertainty budgets shown in Tables 10, 11, 12 and 13, it can be observed that during the calibration of both hydrometers by the traditional method, the alignment “by eye” of the metrologist, at the scale mark of the hydrometer under calibration at the level of the liquid's surface, could not allow us to obtain a better hydrometer resolution, therefore the hydrometer resolution is equal to its scale division. The contribution of the uncertainty by the error due to the resolution *ε_d_* is in this case the dominant uncertainty contribution. It follows that if the hydrometer has a larger scale division (which results in a less accurate hydrometer), then the dominant uncertainty of the hydrometer calibration will be limited by its scale division, as long as the alignment process is done “by eye”.

Putting aside the contribution due to the resolution *ε_d_*, other dominant sources of uncertainty in the hydrometer calibration with the traditional alignment method are the standard deviation of mass determination in the liquid *σm_L_*, the reference liquid surface tension *γ_L_* and the density of the reference liquid *ρ*_L_.

On the other hand, using the vision system and an image processing algorithm to align the mark on the scale to the liquid level, the uncertainty due to the resolution could decrease, because the vision system provides images of the hydrometer immersed in the liquid of such amplitude that divides the scale significantly by about 10 times or more the hydrometer scale division. In this case, resolution was considered as one tenth of the hydrometer scale division. A smaller value has no impact on the combined uncertainty. Also, the contribution due to the standard deviation of the measurements to obtain the mass in the liquid is reduced significantly because the alignment process is repeatable and reproducible, obtaining values of *σm_L_* less than 1 mg while the traditional method (by eye), *σm_L_* could take values until 10 mg.

### 3.4. Alignment Using Image Processing

The image processing algorithm to obtain the alignment of the mark in the calibration scale is presented step by step.

#### Step 1. Image acquisition with the vision system

Here, the image is 1,026 × 1,288 pixels, whose values are between 0 and 255 (grayscale).

#### Step 2. Image normalization

**Figure 12.**
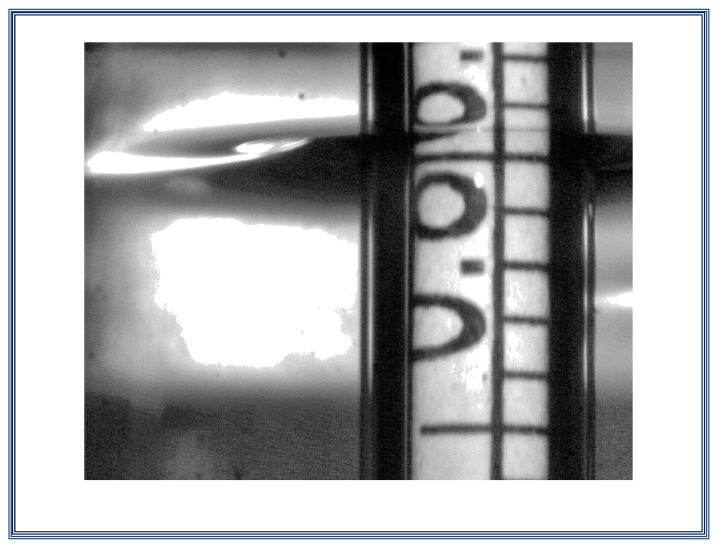
Acquisition and normalization of the image taken with the vision system.

Considering the image in previous step, the image is normalized to take double precision floating point format values between 0 and 1 for the grayscale, as can be seen on Figure 12.

#### Step 3. Negative image

**Figure 13.**
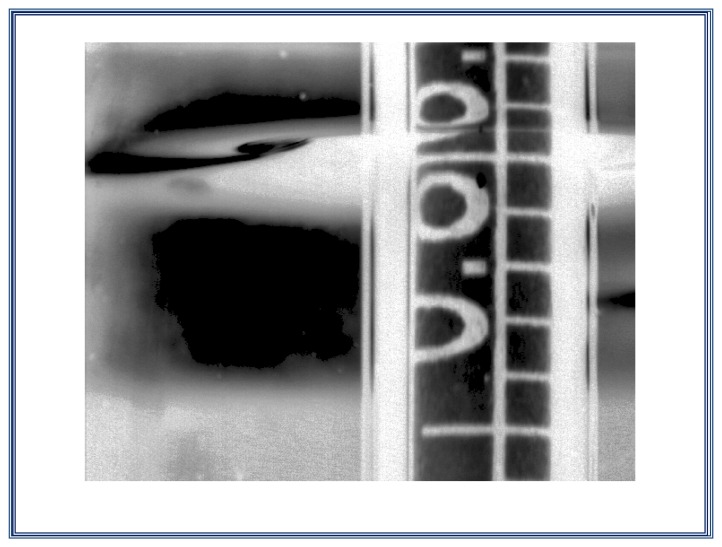
Negative image.

In this step the negative of the normalized image is obtained, in this way, the scale marks, originally black, are displayed in white. This is to approximate a concave downward parabola. Mathematically, a gray level 0 in the original picture, which represents the black color becomes 1 (white on the inverted image) and *vice versa*. Each original gray level becomes its complement in the negative. The negative image is shown in Figure 13.

#### Step 4. Search the region of interest ROI

**Figure 14.**
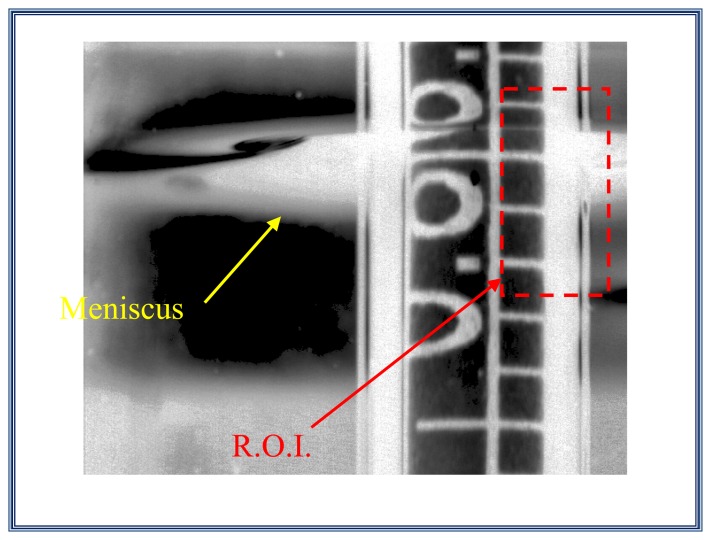
Region of interest (R.O.I.)

In this section, we look for the three marks of scale hydrometer immersed in the liquid; these marks are easy to find by locating the ellipse that is adjacent to the hydrometer shaft (this ellipse is the base of the meniscus that the liquid form around the hydrometer shaft), as can be seen on Figure 14.

#### Step 5. Column vector selection

**Figure 15.**
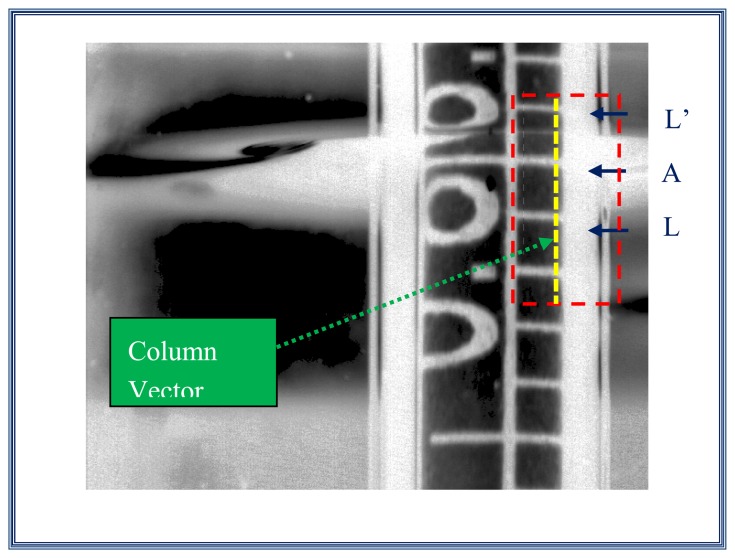
Column vector that contains the three interest marks *L-A-L*'.

**Figure 16.**
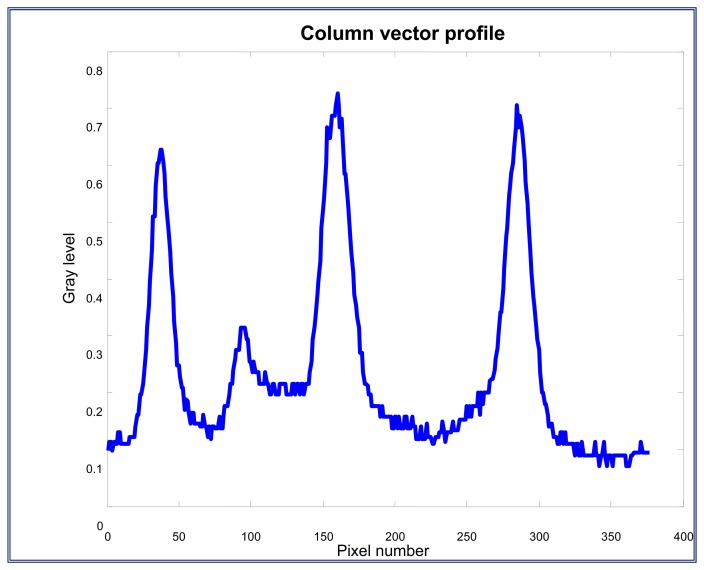
Column vector profile that contains the interest marks.

Here, a column vector is selected. This vector contains the information of the three interest marks, *L-A-L*'. This vector represents the profile or the gray level of the column vector selected and is shown on Figure 15. The profile which includes the interest marks is plotted on Figure 16.

#### Step 6. Mark detection

##### Step 6.1. Column vector filtering

**Figure 17.**
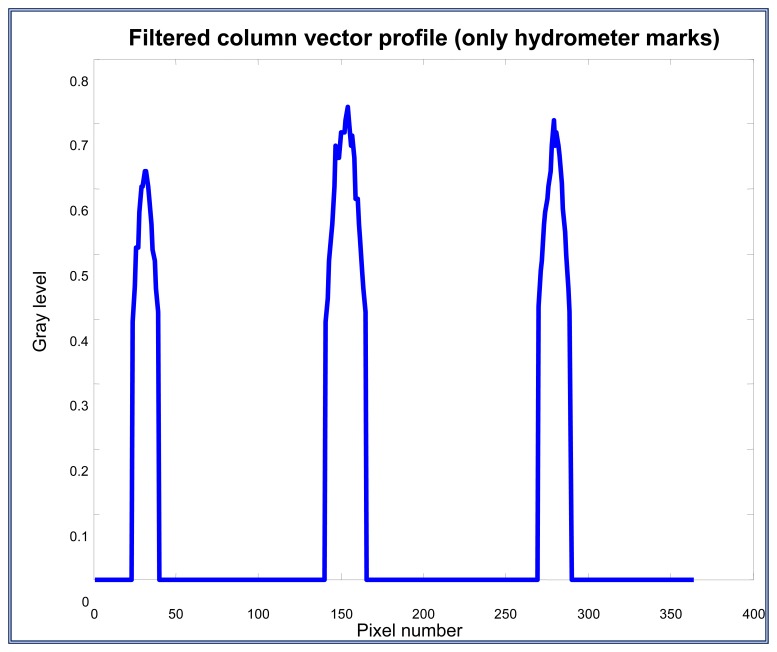
Filtered column vector profile with a 0.6 threshold.

In this process, the column vector is filtered to eliminate noise and leave the information of the three marks only; this is done by applying an appropriate threshold level to get the column vector without noise (generally 0.5 or 0.6). After applying the vector filtering, the profile which includes only the interest marks is shown on Figure 17.

##### Step 6.2. Mark separation and least square adjustment of each mark

**Figure 18.**
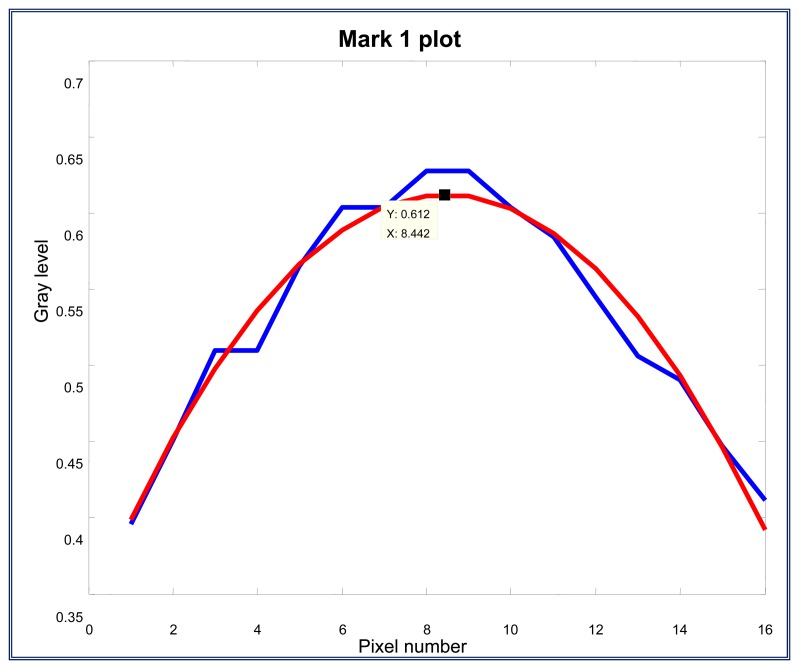
Mark adjustment by using a least square fitting, where *y* = 0.337528 + 0.065020*x* − 0.003851*x*^2^; *x*_max_ = 8.4423; *y*_max_ = 0.6120.

Here, each mark is isolated from the filtered column vector to apply the least square adjustment and fit to a second order polynomial, *y* = *a_0_* + *a_1_x* +*a_2_x^2^*. After the least square adjustment and fitting, the maximum and minimum values of each parabola are determined. In Figure 18, the blue line represents the information of the mark and the least square fitting can be observed on the red line, the second order polynomial equation and the maximum point of each parabola are calculated:

Mark adjustment by using a least square fitting, where *y* = 0.337528 + 0.065020*x* − 0.003851*x*^2^; *x*_max_ = 8.4423; *y*_max_ = 0.6120.

The same procedure is applied to marks 2 and 3. For the second mark, the calculated values are given by; *y*=0.340576+0.055549*x* − 0.002146*x*^2^; *x*_max_=12.9452; *y*_max_=0.7001

For the third mark, the calculated values are given by *y* = 0.343213 + 0.061910*x* − 0.002943*x*^2^; *x*_max_ = 10.5190; *y*_max_ = 0.6688.

#### Step 7. Distances d1 and d2 measurement (in pixels)

**Figure 19.**
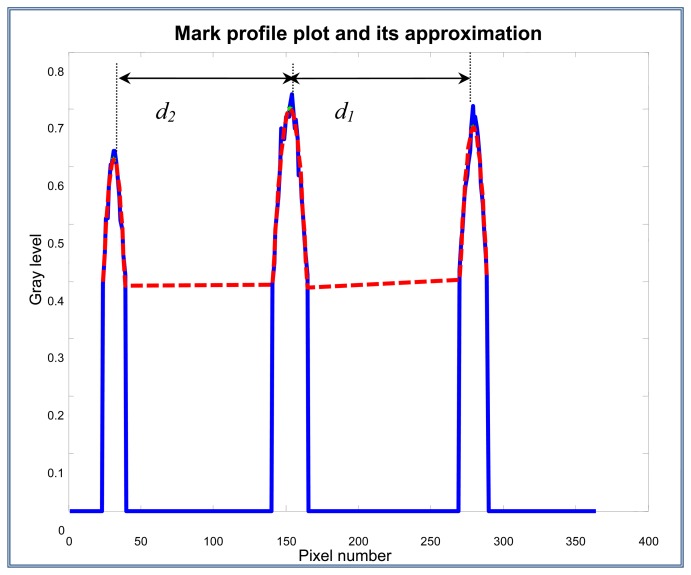
Distance measurement *d_1_* and *d_2_*, and *K_meas_* estimation. *d_2_* = 121.50 pixels; *d_1_* = 126.57 pixels; *K_meas_* = *d_2_*/*d_1_* = 0.96.

Once the parabolas and its maximum values are obtained, the distance *d_1_* is measured (this distance is equal to the distance from the mark *A* to the mark *L*), and the distance *d_2_* (distance from mark *A* to mark *L*'). The distances are considered from the maximum points of the parabolas which were obtained from the gray levels of the filtered column vector. The distances *d_1_* and *d_2_* are shown on Figure 19.

#### Step 8. *K_meas_* determination

*K_meas_* = *d_2_*/*d_1_* = (121.50/126.57) = 0.959 9

#### Step 9. *K_calc_* and *K_meas_* values comparison

*K_calc_* = 0.96 ± 0.03 (*k* = 2) (*k* = 2 represents a confidence level of approximately 95.45% in the uncertainty level from the best estimated *K_calc_* value) and *K_meas_* = 0.96, therefore, it can be stated that mark *A* (corresponding to the mark under calibration, *ρ_n_*) is aligned to the same level than the liquid surface, because *K_meas_* is inside the confidence interval *K_calc_*.

Several tests with its corresponding alignments were carried out, and the summary of the *K_meas_* values are shown by applying the traditional alignment method and the vision system method.

The *K_meas_* values for each alignment method are compared with the *K_calc_* values obtained with the pinhole model approach, for both the high accuracy hydrometer (*d* = 0.1 kg/m^3^) as well as the medium accuracy hydrometer (*d* = 0.5 kg/m^3^) shown in Tables 1 and 2.

**Figure 20.**
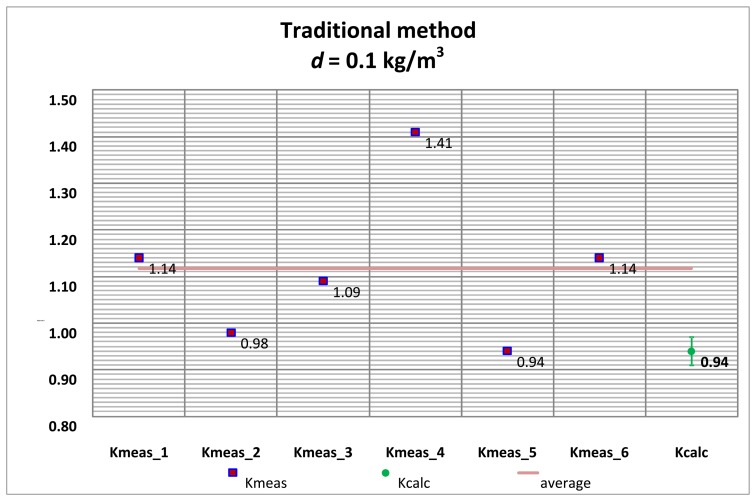
*K_meas_* values. High accuracy hydrometer. Traditional alignment.

**Figure 21.**
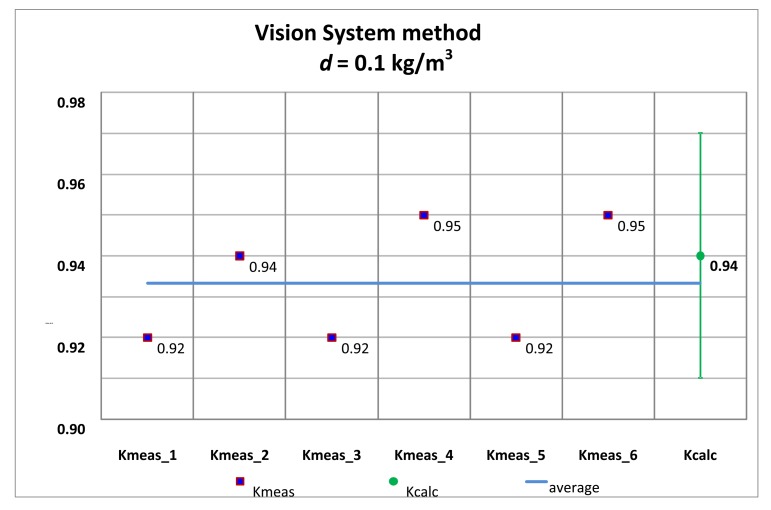
*K_meas_* values. High accuracy hydrometer. Vision system alignment.

**Figure 22.**
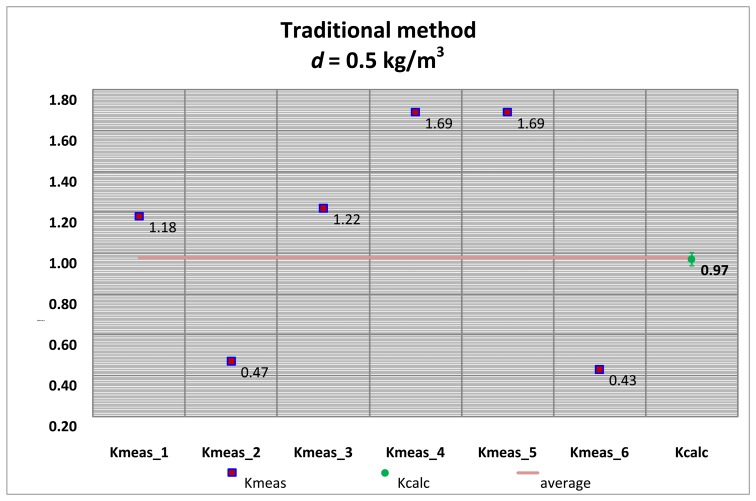
*K_meas_* values. Medium accuracy hydrometer. Traditional alignment.

**Figure 23.**
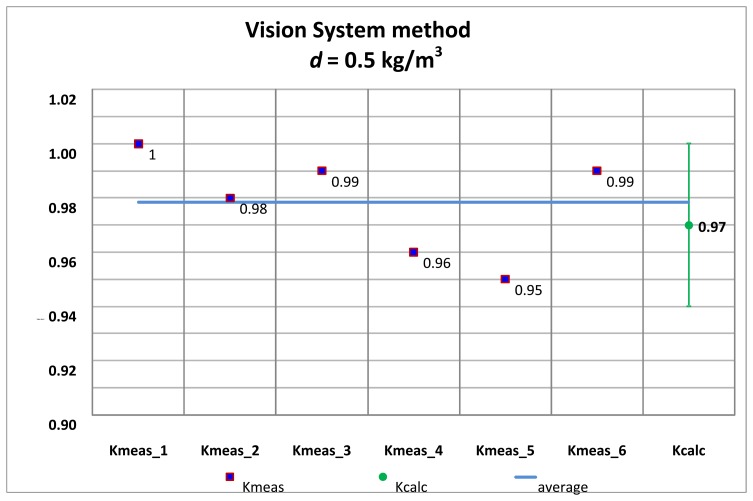
*K_meas_* values. Medium accuracy hydrometer. Vision system alignment.

Figures 20 and 21 correspond to the 0.1 kg/m^3^ hydrometer and Figures 22 and 23 correspond to the 0.5 kg/m^3^ hydrometer. Uncertainty bars of *K_calc_* are equivalent to a 0.03 value with a coverage factor of *k* = 2 for a normal probability distribution.

## 4. Results and Discussion

From Tables 5 and 6, the values of standard deviation in the determination of the mass in the liquid *σm_L_* were 0.12 mg by using the vision system for the alignment of the mark, which in comparison with the traditional alignment system, in the case of the high accuracy hydrometer is 15 times lower, and for the medium accuracy hydrometer is 72 times lower. In the uncertainty budgets the percentage contribution due to this component was reduced from 2.4% to 0.04% in the case of the high accuracy hydrometer calibration (see Tables 10 and 11), and from 21.28% to 0.08% in the case of the medium accuracy hydrometer (see Tables 12 and 13). From the above, it is concluded that the methodology developed in this work to align the mark on the scale calibration of immersion hydrometers at the same level of the surface of the liquid of known density using the vision system and the digital image processing algorithm was able to improve the measurement system, reducing by more than one order the standard uncertainty due to the repeatability of the mass determination in the liquid, *m_L_*.

Moreover, the contribution to the uncertainty due to the resolution of the immersion hydrometer was also reduced significantly, since the conventional alignment method and the resolution of the hydrometer is limited to the division of its scale, so the standard uncertainty error due to the resolution *ε_d_* is equal to $\frac{d}{\sqrt{12}}$, by considering a uniform probability distribution. The hydrometer scale division can be divided into ten units or more, since the amplification of the marks of hydrometer that is achieved with the vision system significantly distinguishes a better resolution compared to the scale division of the hydrometer.

For the case of hydrometers calibrated in this work, the contribution to the standard uncertainty due to the resolution of high accuracy hydrometer, whose scale division is 0.1 kg/m^3^, was reduced from 73.32% to a 2.93% (see Tables 10 and 11), while for medium accuracy hydrometer with a scale division equal to 0.5 kg/m^3^, this contribution was reduced from 72.70% to a 10.76% (see Tables 12 and 13).

In the calibration method of alignment by the vision system, the resolution was taken as one tenth of the hydrometer scale division. A smaller value has no significant impact to the combined uncertainty of calibration which could have many effects in the uncertainty budget [15–19].

Since 2004 the automation or semi-automation of hydrometer calibration has been implemented in other national metrology institutes (NMIs), some of them based on image processing techniques [9–11]. The methodology exposed in this work is based on the approach of Lorefice and Malengo [9] however the vision system exposed in this work has a better spatial resolution, for example, the CCD camera of [9] is 604 H × 576 V pixels whereas CENAM CCD camera is 1,300 H × 1,030 V pixels. Digital images acquired with CENAM vision system allowed us to reduce the resolution of the hydrometer scale division from *d* to *d*/10 or better during the calibration, reducing considerably the uncertainty contribution due to the resolution of hydrometer, this point is not addressed in [9–11]. Moreover, the image processing algorithm for the detection of marks, the perspective errors, and the criteria for the alignment of the scale mark under calibration at the level of the surface liquid are different to the approaches of [9–11]; for example, Lee *et al.* [10] performed a binary threshold on the acquired image of the hydrometer stem and the alignment criteria is to locate the scale mark under calibration in line with the major axis of an ellipse that represents the meniscus formed around the stem in the acquired image. Another remarkable difference is the mechanical system employed to align the hydrometer scale mark to the liquid surface: the CENAM system uses a small motorized sinker allowing a well-controlled and soft adjustment of the level of the liquid without turbulence when the sinker is immersed. Other systems perform the adjustment of the mark by moving the whole system: thermostatic bath, the liquid container and CCD camera.

In metrology, it is mandatory to validate either a new measurement method or a modification with an already validated method [20,21]. In order to validate the methodology proposed in this work based on the aligning mark immersion hydrometer scale under calibration with the vision system and the algorithm of digital image processing [22], two international comparisons in the calibration immersion of hydrometers were carried out.

The first international comparison, identified as SIM.M.D-K4 “*Comparison on the calibration of density hydrometers*”, is a key comparison among national metrology institutes that are members of the Inter-American Metrology System (SIM). In this comparison, CENAM was the pilot laboratory. Other participant countries were: Jamaica, Panama, Chile, Bolivia, Peru, Ecuador, Brazil, Argentina, Costa Rica, Uruguay, Colombia, United States of America and Canada [21]. The second international comparison, identified as SIM.M.D-S1. “*Comparison of the calibration of hydrometers for liquid density determination (bilateral CENAM-INRIM) Supplementary Inter-American Metrology System Comparison (SIM)*”, was a bilateral comparison between CENAM-Mexico and Istituto Nazionale di Metrología di Ricerca Metrologica (INRIM)-Italy [23], and was organized to see the degree of equivalence on the hydrometer calibration between both countries. In both international comparisons, CENAM obtained satisfactory results.

Finally, the methodology proposed in this work for the alignment of the calibration scale mark with the vision system and the image processing technique helps to reduce the relative combined uncertainty calibration of hydrometers from 10^−4^ to 10^−5^.

## Figures and Tables

**Table 1.** Characteristics of the high accuracy immersion hydrometer.

**Parameter**	**Value**
Company:	Steevenson Reevs, LTD.
Serial number:	06/801004
Measurement interval:	800 kg/m^3^ a 810 kg/m^3^
Scale division (*d*):	0.1 kg/m^3^
Calibration point (*ρ_n_*):	805 kg/m^3^
Surface tension of the liquid which is normally used (*γ_x_*):	25 mN/m
Diameter of the shaft (*D*):	4.60 mm
Reference temperature (*t_ref_*):	20 °C
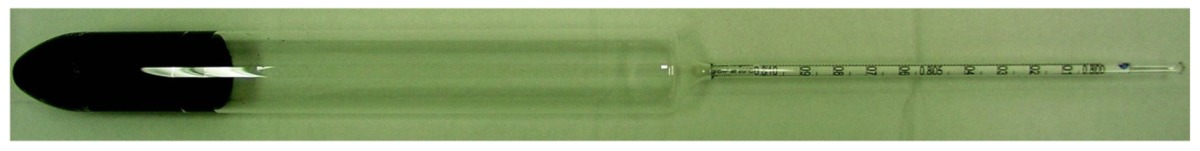	

**Table 2.** Characteristics of the medium accuracy immersion hydrometer.

**Parameter**	**Value**
Company:	Ertco–USA
Serial number:	08482
Measurement interval:	1,000 kg/m^3^ a 1,050 kg/m^3^
Scale division (*d*):	0.5 kg/m^3^
Calibration point (*ρ_n_*):	1025 kg/m^3^
Surface tension of the liquid which is normally used (*γ_x_*):	72 mN/m
Diameter of the shaft (*D*):	5.25 mm
Reference temperature (*t_ref_*):	20 °C
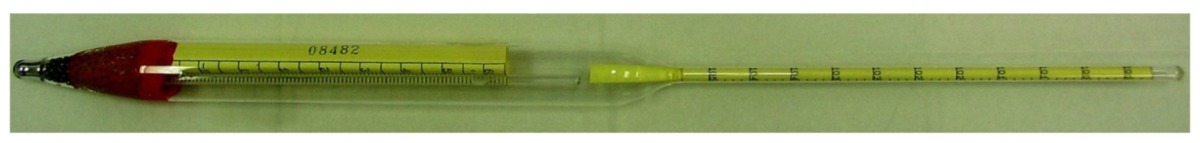	

**Table 3.** *K_calc_* for the high accuracy hydrometer.

**Parameter**	**Value**
Distance between the marks in the hydrometer scale (*y_1_*):	1.2 mm
Vertical distance from the liquid's surface to the camera (*y_2_*):	18 mm
Horizontal from the hydrometer to the camera (*x_T_*):	315 mm
Calculated *K* value (*K_calc_*):	0.94

**Table 4.** *K_calc_* for the medium accuracy hydrometer.

**Parameter**	**Value**
Distance between the marks in the hydrometer scale (*y_1_*):	1.3 mm
Vertical distance from the liquid's surface to the camera (*y_2_*):	37 mm
Horizontal from the hydrometer to the camera (*x_T_*):	315 mm
Calculated *K* value (*K_calc_*):	0.97

**Table 5.** Mass in air of the calibrated hydrometers. The *m_a_* value includes the air pressure corrections and calibration standards. It includes the standard deviation σ*m_a_* of the *m_a_* measurements.

**Hydrometer 06/801004 (High Accuracy)**			**Hydrometer 08482 (Medium Accuracy)**		
	
**Mass in the Air**			**Mass in the Air**		
	*B Hydrometer*	*A Standard*		*B Hydrometer*	*A Standard*
l_1_	110.6582g	110.0000g	l_1_	61.8539g	110.0000g
l_2_	110.6569g	110.0001g	l_2_	61.8547g	110.0001g
l_3_	110.6572g	110.0003g	l_3_	61.8542g	110.0003g
l_4_	110.6582g	110.0001g	l_4_	61.8539g	110.0001g
l_5_	110.6569g	110.0002g	l_5_	61.8547g	110.0002g
l_6_	110.6572g	110.0001g	l_6_	61.8542g	110.0001g
***m*_a_**	**110.644400g**	**110644.00mg**	***m*_a_**	**61.84661g**	**110644.00mg**
**σ *m*_a_**	**0.00065g**	**0.65mg**	**σ *m*_a_**	**0.00036g**	**0.65mg**

**Table 6.** Mass in the liquid of the high accuracy hydrometer. The *m_L_* values include the air pressure corrections and the calibrated standards. It includes the standard deviation σ*m_L_* of the *m_L_* measurements.

**Hydrometer 06/801004 (High Accuracy)**			**Hydrometer 08482 (Medium Accuracy)**		
	
**Traditional Method Mass in the Liquid at Point 805 kg/m^3^**			**Method Using the Vision System Mass in the Liquid at Point 805 kg/m^3^**		
	*B Hydrometer*	*A Standard*		*B Hydrometer*	*A Standard*
l_1_	5.0147g	5.0001g	l_1_	5.0149g	5.0001g
l_2_	5.0138g	5.0000g	l_2_	5.0148g	4.9999g
l_3_	5.0116g	5.0001g	l_3_	5.0150g	5.0000g
l_4_	5.0150g	5.0003g	l_4_	5.0150g	4.9999g
l_5_	5.0126g	5.0002g	l_5_	5.0149g	4.9999g
l_6_	5.0164g	5.0003g	l_6_	5.0149g	5.0000g
***m*_L_**	**5.01324g**	**5013.24mg**	***m*_L_**	**5.01434g**	**5014.34mg**
**σ *m*_L_**	**0.00168g**	**1.68mg**	**σ *m*_L_**	**0.00011g**	**0.11mg**

**Table 7.** Mass in the liquid of the medium accuracy hydrometer. The *m_L_* values include the air pressure corrections and the calibrated standards. It includes the standard deviation σ*m_L_* of the *m_L_* measurements.

**Hydrometer 08482 (Medium Accuracy)**			**Hydrometer 08482 (Medium Accuracy)**		
	
**Traditional Method Mass in the Liquid at Point 1,025 kg/m^3^**			**Method Using the Vision System Mass in the Liquid at Point 1,025 kg/m^3^**		
	*B Hydrometer*	*A Standard*		*B Hydrometer*	*A Standard*
l_1_	15.4332g	15.0001g	l_1_	15.4367g	15.0003g
l_2_	15.4425g	15.0000g	l_2_	15.4368g	15.0002g
l_3_	15.4317g	15.0001g	l_3_	15.4367g	15.0004g
l_4_	15.4268g	15.0001g	l_4_	15.4366g	15.0003g
l_5_	15.4219g	15.0002g	l_5_	15.4367g	15.0003g
l_6_	15.4440g	15.0003g	l_6_	15.4368g	15.0004g
***m*_L_**	**15.43122g**	**15431.22mg**	***m*_L_**	**15. 43459g**	**15434.59mg**
**σ *m*_L_**	**0.00868g**	**8.68mg**	**σ *m*_L_**	**0.00012g**	**0.12mg**

**Table 8.** Calibration results of the high accuracy hydrometer.

**Method**	**Nominal Value *ρ****_n_*	**Density* ρ****_x_*	**Correction *C****_ρn_*	**Unc. (*k*=2) *U* (*C****_ρn_***)**
Traditional	805	805.034 kg/m^3^	0.034 kg/m^3^	0.067 kg/m^3^
Vision System	805	805.041 kg/m^3^	0.041 kg/m^3^	0.034 kg/m^3^

Hydrometer 06/801004.

**Table 9.** Calibration results of the medium accuracy hydrometer.

**Method**	**Nominal Value *ρ****_n_*	**Density *ρ****_x_*	**Correction *C****_ρn_*	**Unc. (*k*=2) *U* (*C****_ρn_***)**
Traditional	1025	1024.947 kg/m^3^	−0.053 kg/m^3^	0.339 kg/m^3^
Vision System	1025	1025.024 kg/m^3^	0.024 kg/m^3^	0.088 kg/m^3^

Hydrometer 08482.

**Table 10.** Uncertainty budget − Traditional method − *d* = 0.1 kg/m^3^.

**Uncertainty budget−Traditional Method**					

	**Ci**	**Std. Unc.**	**Contrib. (kg/m^3^)**	**Variance (kg^2^/m^3^)**	**%**
δρx/δρL=	1.047412213	1.06E–02	0.0111	1.240E–04	10.91
δρx/δρa1=	−0.047412447				
δρx/δma=	−344.8652533				
δρx/δmL=	7609.201784				
δρx/δtL=	0.007970273	0.01	0.0001	6.353E–09	0.00
δρx/δta=	1.92822E–05	0.2	0.0000	1.487E–11	0.00
δρx/δγL=	−11.73150782	1.05E–03	−0.0123	1.517E–04	13.35
**δρx/δd=**	−**1**	**0.028867513**	**−0.0289**	**8.33E–04**	**73.32**
δma/δmp1=	0.999879272	3.35E–08	0.0000	1.334E–10	0.00
δma/δρa1=	−0.00001396	0.00077023	0.0000	1.334E–09	0.00
δma/δρp1=	1.69E–09	79.31	0.0000	2.125E–09	0.00
δma/δΔm1=	1	2.64155E–07	−0.0001	8.299E–09	0.00
δma/δd=	−1	2.88675E–08	0.0000	9.911E–11	0.00
δmL/δmp2=	0.999879272	4.50E–08	0.0003	1.172E–07	0.01
δmL/δρa2=	−0.000000621	0.000762157	0.0000	2.006E–09	0.00
δmL/δρp2=	7.309775E–11	80.52	0.0000	2.006E–09	0.00
**δmL/δΔm2=**	**1**	**6.86254E–07**	**0.0052**	**2.727E–05**	**2.40**
δmL/δd=	−1	2.88675E–08	−0.0002	4.825E–08	0.00
δmliq/δCg=	1				
δCg/δΔh=	−1.57757E–09	0.01	0.0000	1.441E–14	0.00
**Unc. (k=2)**	**0.067 kg/m^3^**				

**Table 11.** Uncertainty budget − Vision System method − *d* = 0.1 kg/m^3^.

**Uncertainty budget**-**Vision System**					

	**Ci**	**Std. Unc.**	**Contrib. (kg/m^3^)**	**Variance (kg^2^/m^3^)**	**%**
δρx/δρL=	1.047423126	1.06E–02	0.0111	1.240E–04	43.60
δρx/δρa1=	−0.047412335				
δρx/δma=	−344.9478424				
δρx/δmL=	7609.353397				
δρx/δtL=	0.007970356	0.01	0.0001	6.353E–09	0.00
δρx/δta=	1.92823E−05	0.2	0.0000	1.487E–11	0.00
δρx/δγL=	−11.73174157	1.05E–03	−0.0123	1.517E–04	53.36
**δρx/δd**=	−**1**	**0.002886751**	−**0.0029**	**8.333E**–**06**	**2.93**
δma/δmp1=	0.999879272	3.35E–08	0.0000	1.335E–10	0.00
δma/δρa1=	−0.00001396	0.00077023	0.0000	1.334E–09	0.00
δma/δρp1=	1.69E−09	79.31	0.0000	2.126E–09	0.00
δma/δΔm1=	1	2.64155E–07	−0.0001	8.303E–09	0.00
δma/δd=	−1	2.88675E–08	0.0000	9.916E–11	0.00
δmL/δmp2=	0.999879272	4.50E–08	0.0003	1.172E–07	0.04
δmL/δρa2=	−0.000000621	0.000762481	0.0000	1.298E–11	0.00
δmL/δρp2=	7.30987E–11	80.52	0.0000	2.006E–09	0.00
**δmL/δΔm2=**	**1**	**4.40959E**–**08**	**0.0003**	**1.126E–07**	**0.04**
δmL/δd=	−1	2.88675E–08	−0.0002	4.825E–08	0.02
δmliq/δCg=	1				
δCg/δΔh=	−1.57757E–09	0.01	0.0000	1.441E–14	0.00
**Unc. (k = 2)**	**0.034 kg/m^3^**				

**Table 12.** Uncertainty budget − Traditional method − *d* = 0.5 kg/m^3^.

**Uncertainty budget-Traditional Method**					

	**Ci**	**Std. Unc.**	**Contrib. (kg/m^3^)**	**Variance (kg^2^/m^3^)**	**%**
δρx/δρL=	1.333875213	1.06E–02	0.0142	2.011E–04	0.70
δρx/δρa1=	−0.333877216				
δρx/δma=	−5523.532137				
δρx/δmL=	22039.93249				
δρx/δtL=	0.01015011	0.01	0.0001	1.030E–08	0.00
δρx/δta=	2.19729E–05	0.2	0.0000	1.931E–11	0.00
δρx/δγL=	−37.16576354	1.05E–03	−0.0390	1.523E–03	5.31
**δρx/δd=**	**−1**	**0.144337567**	**−0.1443**	**2.083E–02**	**72.70**
δma/δmp1=	0.99987947	3.35E–08	−0.0002	3.423E–08	0.00
δma/δρa1=	−0.000007858	0.00077029	−0.0003	6.614E–08	0.00
δma/δρp1=	9.47E–10	78.90	−0.0004	1.704E–07	0.00
δma/δΔm1=	1	1.47573E–07	−0.0008	6.644E–07	0.00
δma/δd=	−1	2.88675E–08	0.0002	2.542E–08	0.00
δmL/δmp2=	0.999880825	4.50E–08	0.0010	9.843E–07	0.04
δmL/δρa2=	−0.000001881	0.000769758	0.0000	1.018E–09	0.00
δmL/δρp2=	2.24168E–10	79.74	0.0004	1.552E–07	0.00
**δmL/δΔm2=**	**1**	**3.54287E–06**	**0.0781**	**6.097E–03**	**21.28**
δmL/δd=	−1	2.88675E–08	−0.0006	4.048E–07	0.00
δmliq/δCg=	1				
δCg/δΔh=	−4.73272E–09	0.01	0.0000	1.088E–12	0.00
**Unc. (k=2)**	**0.339 kg/m^3^**				

**Table 13.** Uncertainty budget – Vision System method – *d* = 0.5 kg/m^3^.

**Uncertainty budget-Vision System**					

	**Ci**	**Std. Unc.**	**Contrib. (kg/m^3)^**	**Variance (kg^2^/m^3^)**	**%**
δρx/δρL=	1.333976871	1.06E–02	0.0142	2.011E–04	10.38
δρx/δρa1=	−0.333978901				
δρx/δma=	−5525.645555				
δρx/δmL=	22043.33278				
δρx/δtL=	0.010150884	0.01	0.0001	1.030E–08	0.00
δρx/δta=	2.19739E–05	0.2	0.0000	1.931E–11	0.00
δρx/δγL=	−37.17149742	1.05E–03	−0.0390	1.523E–03	78.66
**δρx/δd**=	**−1**	**0.014433757**	**−0.0144**	**2.083E–04**	**10.76**
δma/δmp1=	0.99987947	3.35E–08	−0.0002	3.426E–08	0.00
δma/δρa1=	−0.000007858	0.00077029	−0.0003	6.618E–08	0.00
δma/δρp1=	9.47E–10	78.90	−0.0004	1.705E–07	0.01
δma/δΔm1=	1	1.47573E–07	−0.0008	6.649E–07	0.03
δma/δd=	−1	2.88675E–08	0.0002	2.544E–08	0.00
δmL/δmp2=	0.999880819	4.50E–08	0.0010	9.837E–07	0.05
δmL/δρa2=	−0.000001881	0.000769742	0.0000	1.019E–09	0.00
δmL/δρp2=	2.2418E–10	79.74	0.0004	1.553E–07	0.01
**δmL/δΔm2**=	**1**	**5.47723E–08**	**0.0012**	**1.458E–06**	**0.08**
δmL/δd=	−1	2.88675E–08	−0.0006	4.049E–07	0.00
δmliq/δCg=	1				
δCg/δΔh=	−4.73272E–09	0.01	0.0000	1.088E–12	0.00
**Unc. (k=2)**	**0.088 kg/m^3^**				

## References

[1] (1982). ISO-4805 Laboratory Glassware—Thermo-Alcoholometers and Alcohol-Thermohydrometers.

[2] (1979). ISO-4801 Glass Alcoholometers and Alcohol Hydrometers not Incorporating a Thermometer.

[3] (1985). OIML R44 Alcoholometers and Alcohol Hydrometers and Thermometers for Use in Alcoholometry.

[4] (1983). IS-7324 Indian Standard Specification for Brix Hydrometers.

[5] (1974). ISO-2449 Milk and Liquid Milk Products–Density Hydrometers for Use in Products with a Surface Tension of Approximately 45 mN/m.

[6] (1984). ISO-3993 Liquefied Petroleum Gas and Light Hydrocarbons–Determination of Density or Relative Density–Preasure Hydrometer Method.

[7] Gupta S.V. (2003). Practical density measurement and hydrometry. Meas. Sci. Technol..

[8] Cuckow F.W. (1949). A new method of high accuracy for the calibration of reference standard hydrometers. J. Soc. Chem. Ind..

[9] Lorefice S., Malengo A. (2004). An image processing approach to calibration of hydrometers. Metrologia.

[10] Lee Y.J., Chang K.H., Chon J.C., Oh C.Y. (2004). Automatic alignment method for calibration of hydrometers. Metrologia.

[11] Aguilera J., Wright J. D., Bean Vern E. (2008). Hydrometer calibration by hydrostatic weighing with automated liquid surface positioning. Meas. Sci. Technol..

[12] Lorefice S., Malengo A. (2006). Calibration of hydrometers. Meas. Sci. Technol..

[13] (2012). BIPM, IEC, IFCC, ILAC, ISO, IUPAC, IUPAP and OIML JCGM 200: 2012 International Vocabulary of Metrology–Basic and General Concepts and Associated Terms (VIM).

[14] (2008). BIPM, IEC, IFCC, ILAC, ISO, IUPAC, IUPAP and OIML JCGM-100: 2008 Evaluation of Measurement Data–Guide to the Expression of Uncertainty in Measurement.

[15] Valcu A. (2007). Calibration of nonautomatic weighing instruments. Measurements.

[16] Tasic T., Grottker U. (2006). An overview of guidance documents for software in metrological applications. Comput. Stand. Interf..

[17] Heinonen M., Sampo S. (2003). The effect of density gradients on hydrometers. Measur. Sci. Technol..

[18] Peña L.M., Becerra L.O. Evaluation of the Uncertainty due to Instability of the Measurement Standards Involved in a Measurement Process.

[19] Picard A., Davis R.S., Gläser M., Fujii K. (2008). Revised formula for the density of moist air (CIPM-2007). Metrologia.

[20] Lorefice S., Heinonen M., Madec T. (2000). Bilateral comparisons of hydrometer calibrations between the IMGC-LNE and the IMGC-MIKES. Metrologia.

[21] Becerra L.O. (2009). Final report of comparison of the calibrations of hydrometers for liquid density determination between SIM laboratories: SIM.M.D-K4. Metrologia.

[22] Peña L.M., Pedraza J.C., Becerra L.O., Galvan C.A. A New Image Processing System for Hydrometers Calibration Developed at CENAM.

[23] Becerra L.O., Lorefice S. (2009). Report of the bilateral comparison of the calibrations of hydrometers for liquid density determination between CENAM–Mexico and INRIM–Italy: SIM.M.D-S1. Metrologia.

